# Robust slice-level stroke classification in non-contrast head CT via structural consistency regularization and counterfactual suppression

**DOI:** 10.3389/fbioe.2026.1799191

**Published:** 2026-06-08

**Authors:** Dong Xu, Qing Yao, Lifeng Qian

**Affiliations:** 1 Auckland Tongji Rehabilitation Medical Equipment Research Center, Tongji Zhejiang College, Jiaxing, China; 2 School of Mechanical Engineering, Tongji University, Shanghai, China; 3 Department of Rehabilitation, Jiaxing Hospital of Traditional Chinese Medicine Affiliated to Zhejiang Chinese Medical University, Jiaxing, China

**Keywords:** brain stroke, consistency regularization, counterfactual suppression, CT image classification, neurorehabilitation

## Abstract

**Background:**

Stroke is a time-critical neurological condition with abrupt onset, rapid progression, and a narrow treatment window. In emergency settings, non-contrast head CT is widely used for initial screening and preliminary subtyping, but single-slice CT interpretation remains challenging because lesions often show low contrast, blurred boundaries, diverse morphologies, and interference from skull-related structures and imaging artifacts.

**Methods:**

We propose a slice-level stroke CT classification framework that integrates structural consistency regularization, counterfactual suppression, and multi-branch feature refinement. The consistency branch enforces representation stability under perturbations and structure-preserving transformations, while the counterfactual branch attenuates spurious cues and refines branch-wise evidence before confidence-aware aggregation.

**Results:**

Comparative experiments on two stroke CT slice datasets show that the proposed method achieves the best overall performance in terms of Accuracy, Precision, Recall, F1, and AUC. On the primary dataset, it reaches Acc = 0.9823, Precision = 0.9766, Recall = 0.9740, F1 = 0.9805, and AUC = 0.9982. On the secondary dataset, it reaches Acc = 0.9652, Precision = 0.9688, Recall = 0.9773, F1 = 0.9698, and AUC = 0.9989.

**Conclusion:**

These results indicate that the proposed method can more reliably identify stroke-discriminative evidence under low-contrast and high-interference conditions in the single-slice setting, thereby providing more robust support for emergency stroke triage, preliminary diagnosis, and lesion-aware imaging feedback for intelligent neurorehabilitation applications.

## 1 Introduction

Stroke remains a major public health challenge worldwide and is among the leading causes of death and long-term disability, with sudden onset, rapid progression, and a limited therapeutic window GBD 2019 Stroke Collaborators (2021), Li et al. (2021). Its high mortality, persistent disability burden, and prolonged rehabilitation expenditure place considerable pressure on patients, families, and healthcare systems, making early and dependable stroke identification a clinically and socially important task. In routine emergency practice, CT is still one of the most commonly used imaging tools for stroke screening and preliminary subtype assessment because it offers a practical combination of rapid acquisition, reasonable cost, and broad accessibility Zerna et al. (2016), Song et al. (2019). However, stroke-related abnormalities on CT slices are often difficult to recognize because they may appear with weak contrast, indistinct margins, and heterogeneous morphologies, while also being easily affected by bony structures, imaging noise, and anatomical variation. These factors increase radiologists’ interpretation burden and elevate the risk of diagnostic error. Accordingly, developing an intelligent method that can identify stroke-related abnormalities more stably and reliably at the single-slice level is of substantial clinical value for triage, assisted diagnosis, and treatment planning Gao et al. (2017), Shen et al. (2025).

Non-contrast head CT is widely used in emergency stroke evaluation because it provides an effective balance between diagnostic utility, examination efficiency, safety, and practical availability. Compared with head X-ray, non-contrast head CT can directly visualize intracranial structures and reveal clinically important abnormalities such as hemorrhage, mass effect, and early density changes, whereas head X-ray offers very limited value for assessing acute intracranial stroke lesions. Compared with MRI, although MRI is more sensitive to certain early ischemic changes, its application in hyperacute emergency scenarios is often restricted by longer acquisition time, higher cost, reduced availability, and stricter examination requirements. By contrast, non-contrast head CT does not require contrast injection, can be performed rapidly within standard emergency workflows, and is therefore commonly adopted as the first-line imaging modality for initial stroke screening, hemorrhage exclusion, and early triage decision-making Zerna et al. (2016), Song et al. (2019). For this reason, the present study specifically focuses on single-slice non-contrast head CT rather than other imaging modalities.

Despite these practical advantages, intelligent recognition from single-slice non-contrast head CT remains technically challenging. Although deep learning has achieved considerable success in medical image analysis, stroke classification on head CT slices still faces several unresolved difficulties Karthik et al. (2020). First, the model may capture spurious lesion-irrelevant correlations, such as skull-edge enhancement under bone-window effects or texture bias introduced by device and reconstruction differences, which may appear useful during training but can lead to high-confidence errors at inference time. Second, slice-level supervision alone is often insufficient to force the network to attend consistently to true lesion evidence, which may result in attention drift and unstable predictions Gaidhani et al. (2019). Third, augmentation perturbations, threshold variation, and hard-case distributions may substantially alter the Precision-Recall trade-off during training, thereby weakening robustness in clinically important operating regions, such as high-recall or low-false-alarm scenarios Chavva et al. (2022). To summarize these issues more clearly, Table 1 presents a concise comparison between the major challenges and the objectives of this work 1. To address the above problems, this paper investigates single-slice non-contrast head CT stroke classification and develops a unified framework that combines structure-constrained learning with counterfactual intervention, aiming to improve discriminative stability and make error behavior more controllable under the same data distribution. More specifically, consistency constraints are imposed on predictions under perturbations and structure-preserving transformations so that the model can learn more stable discriminative representations and reduce attention drift. Meanwhile, a controllable counterfactual intervention mechanism is introduced to suppress potentially misleading evidence and encourage the model to base its decisions on more clinically meaningful lesion-related features.

**TABLE 1:** Key challenges in stroke CT slice recognition and the objectives of this work.

Challenge	Objective in this work
Spurious correlation reliance	Suppress non-lesion cues
Decision instability	Strengthen consistency constraints
High-confidence misclassification	Reduce error risk

The main contributions of this paper, in correspondence with the above challenges and their clinical implications for stroke diagnosis and treatment, are summarized as follows:

1. We develop a unified framework for single-slice stroke CT classification that integrates consistency regularization with counterfactual suppression and multi-branch feature refinement. Through the joint action of these components, the method improves decision stability, suppresses lesion-irrelevant interference, and enhances lesion-relevant evidence, thereby alleviating unstable prediction and attention drift in non-contrast head CT interpretation.
2. We design a confidence-aware counterfactual refinement strategy and conduct sensitivity analysis on key hyperparameters. The results verify empirical findings such as the effectiveness of moderate intervention strength and the existence of a relatively stable step-size range, providing reproducible parameter settings for practical application while directly reducing excessive reliance on non-lesion but highly correlated cues.
3. We carry out systematic evaluation and error analysis on two datasets, including inspection of high-confidence misclassifications and comparison of discriminative behavior at both the score and curve levels. Both quantitative results and behavioral evidence show that the proposed method can reduce error risk and improve clinically relevant discriminative reliability, thereby offering more dependable support for emergency stroke screening, preliminary diagnosis, and timely treatment decision-making.

## 2 Related work

### 2.1 Brain stroke classification on CT: traditional machine learning and early deep learning

Before deep learning became the dominant paradigm, CT-based intelligent stroke analysis was mainly built upon a conventional pipeline consisting of image preprocessing, handcrafted feature extraction, and traditional classification models. Research in this stage largely emphasized interpretable imaging descriptors and relatively stable rule-based analytical procedures. Nowinski et al. proposed an automatic framework for the detection, localization, and volume estimation of ischemic infarcts on non contrast CT, emphasizing rule based candidate region generation and quantitative measurements. Their study demonstrated the early feasibility of automated ischemic lesion analysis, but the framework remained sensitive to image noise, window width and window level variation, and poorly defined lesion boundaries, which limited its robustness and cross-center generalization ability Nowinski et al. (2013). Kuang et al. introduced machine learning for automated ASPECTS scoring, partially embedding a clinical scoring protocol into a learnable computational framework and improving process consistency. Even so, the overall performance still depended heavily on the reliability of segmentation and localization stages, as well as on distributional consistency across the data, making the method vulnerable to uncertainty accumulation when dealing with subtle low-contrast ischemic signs Kuang et al. (2019). Qiu et al. further focused on early infarct detection in acute stroke and explored machine learning based discrimination strategies on non contrast CT. Although their results confirmed that traditional methods can be useful for specific clinical subtasks, they also exposed the limited representation capacity of handcrafted approaches and their weak sensitivity to subtle density variation, which may lead to persistent missed detections in early, occult, and boundary-blurred lesions Qiu et al. (2020). Overall, these studies established the basic feasibility of CT-based intelligent stroke analysis, yet their strong dependence on manually designed features and handcrafted processing procedures restricted their adaptability and robustness in complex clinical environments.

With the rise of end-to-end learning, research gradually shifted from handcrafted feature engineering to data-driven representation learning based on convolutional neural networks and related deep architectures. Chilamkurthy et al. proposed a deep learning system for detecting critical abnormalities on head CT, demonstrating the potential of deep models in multi class emergency finding recognition, but the retrospective design of the study and the gap between experimental conditions and real clinical workflows also revealed unresolved issues in threshold selection, false-positive control, and cross-device distribution shift Chilamkurthy et al. (2018). Ginat analyzed CT cases flagged by deep learning software and pointed out potential mismatches between model outputs and radiologists’ diagnostic focus, highlighting that strong detection capability does not automatically translate into interpretable and clinically verifiable evidence chains Ginat (2020). At the data level, Flanders et al. summarized the construction and collaborative framework of the RSNA 2019 intracranial hemorrhage challenge dataset, promoting standardized benchmarks, while also noting that discrepancies between challenge data and real clinical distributions may impair model robustness to atypical cases and diverse scanning protocols Flanders et al. (2020). Focusing on acute intracranial hemorrhage, Wang et al. demonstrated that deep networks can achieve strong performance in hemorrhage subtype classification, but they also pointed out that class imbalance and label noise may still impair generalization performance Wang et al. (2021). For early ischemic core identification, Pan et al. employed residual networks to enhance fine grained representation learning, although single-slice or local-field modeling may still neglect broader spatial continuity and global brain context Pan et al. (2021). Addressing the challenge of early invisibility in ischemia, Lu et al. proposed a two stage deep learning model that progressively amplifies weak cues, which helped reduce missed detections but also introduced greater cascade error propagation risk and increased training and deployment complexity Lu et al. (2022). Gauriau et al. reported high accuracy of a head CT deep model for early infarct estimation, demonstrating the promise of end-to-end learning for quantitative assessment, while also indicating that model stability still requires validation on multicenter and heterogeneous populations Gauriau et al. (2023).More recent studies have further moved toward clinically grounded evaluation. Yun et al. validated an acute intracranial hemorrhage detection algorithm through a randomized clinical trial, emphasizing that model performance must translate into tangible gains in diagnostic timeliness and workflow efficiency, while imposing stricter requirements on regulation, bias control, and prospective quality assurance Yun et al. (2023). Similarly, Savage et al. conducted a prospective evaluation of AI triage on non contrast head CT, showing improvements in triage efficiency but also raising concerns about workload implications and accountability arising from false positives and false negatives. Taken together, these studies indicate that deep learning has substantially advanced stroke-related CT analysis; however, important challenges remain in reliability, controllability, generalization, and practical clinical usability Savage et al. (2024).

### 2.2 Advanced deep learning for stroke CT: multi-window, 3D context, and robust generalization

Another important line of research in stroke CT analysis has focused on more explicit utilization of imaging priors, particularly window width/window level information, together with multi-view representation strategies. Karki et al. proposed a trainable CT window neural network that integrates multiple window settings into a unified learning framework to improve intracranial hemorrhage detection, marking a transition from manually selected fixed window parameters to learnable data-driven window optimization, although the learned strategy may still remain coupled to specific scanning protocols and reconstruction kernels Karki et al. (2020). Songsaeng et al. further proposed an HU to RGB transformation with automatic window selection for non contrast CT hemorrhage classification, which reduces the complexity of input construction but may also risk losing subtle information in low-contrast or mixed-lesion scenarios Songsaeng et al. (2025). Teneggi et al. introduced examination level supervision for head CT hemorrhage detection to reduce the cost of slice level annotation, yet this weakly supervised paradigm may be less sensitive to small, sparse, or poorly defined lesions Teneggi et al. (2023). Yeo et al. systematically evaluated multiple training and data processing strategies for intracranial hemorrhage detection, showing that engineering-level optimization is important for deployment, while also indicating that the transferability of such gains across datasets and hospital environments still requires rigorous external validation Yeo et al. (2023). Overall, these studies suggest that multi-window modeling and supervision redesign can improve diagnostic performance, but their effectiveness remains closely tied to data consistency, scanning conditions, and deployment context.

A further major direction has been the incorporation of volumetric information and the strengthening of model generalization for real clinical application. Burduja et al. proposed a CNN LSTM framework on 3D CT scans for hemorrhage detection and subtype classification, capturing inter slice dependencies through sequential aggregation, but such hybrid architectures also impose greater demands on inference efficiency and memory consumption Burduja et al. (2020). Singh et al. presented a shallow 3D CNN for acute brain hemorrhage recognition, which improves deployability but sacrifices part of the representational capacity needed for more complex discrimination tasks Singh et al. (2020). Yu et al. proposed a robust deep segmentation approach for intracerebral hemorrhage hematoma volume estimation, emphasizing the value of volumetric metrics for treatment decisions, while also revealing the sensitivity of segmentation performance to annotation consistency and lesion-boundary definition Yu et al. (2022). Hu et al. developed a hybrid 2D and 3D UNet for aneurysmal subarachnoid hemorrhage detection and quantification with external validation, demonstrating enhanced generalization at the expense of increased training and deployment complexity Hu et al. (2023). Ostmeier et al. proposed random expert sampling for acute ischemic segmentation on non contrast CT to model inter expert variability, although this strategy relies on long-term access to multiple expert annotations, which may be difficult to maintain in large-scale practice Ostmeier et al. (2025). Lin et al. introduced the parameter efficient DGA3 Net for ASPECTS assessment on non contrast CT, providing a more deployment-friendly solution for resource-limited scenarios Lin et al. (2023). Lee et al. conducted a clinical evaluation of automated ASPECTS scoring models and underscored the need for real workflow validation beyond offline metrics Lee et al. (2024). Chavoshi et al. further showed through real world evaluation that external distribution shifts, case mix variation, and workflow differences can substantially affect model performance Chavoshi et al. (2025). In summary, recent advances have substantially promoted stroke-related CT analysis, but robust generalization, dependence on annotation quality, and practical clinical applicability are still not fully resolved.

## 3 Methods

### 3.1 Problem definition

Given a dataset $D={\left\{\left(x_{i},y_{i}\right)\right\}}_{i=1}^{N}$ for the head CT stroke classification task, where $x_{i}\in R^{T\times H\times W}$ denotes an input composed of T adjacent slices (or volumetric data) and $y_{i}\in \left\{1,\ldots ,K\right\}$ represents the corresponding stroke category label (e.g., normal, hemorrhagic, ischemic), our objective under the standard supervised learning setting is to learn a Swin-Transformer based classifier $f_{\theta }:R^{T\times H\times W}\to \Delta^{K}$ by minimizing the empirical risk $\frac{1}{N}\sum_{i=1}^{N}\ell \left(f_{\theta }\left(x_{i}\right),y_{i}\right)$ with respect to the parameters θ , where ℓ(⋅,⋅) denotes the classification loss function. Building upon this formulation and targeting improvements in both classification accuracy and predictive stability, we further introduce counterfactual learning within the same training distribution to construct contrastive representations that suppress non causal correlated features, and impose domain invariant consistency regularization under feature perturbations and structural constraints to reinforce prediction consistency, thereby enhancing the model’s robust discriminative capability against complex lesion morphology, variations in window width and window level, and noise perturbations.

Compared with existing CT-based stroke recognition methods, the advantages of the proposed framework can be summarized in three main aspects. Sahoo et al. primarily addressed the automatic detection of early ischemic lesions on non-contrast CT using deep learning, with the emphasis placed on lesion recognition under a conventional supervised learning paradigm; however, their method did not explicitly enforce prediction consistency under perturbations or address the influence of spurious correlations during decision-making Sahoo et al. (2022). Ayoub et al. developed a vision transformer framework for brain stroke assessment based on multi-slice CT classification and localization, where the main focus was on cross-slice representation learning and localization modeling rather than on improving decision stability within the same data distribution Ayoub et al. (2023). Alis et al. proposed a convolutional-recurrent architecture with an attention mechanism for intracranial hemorrhage detection on non-contrast head CT, demonstrating the value of sequential aggregation and attention-based enhancement, yet still lacking an explicit strategy for suppressing non-causal evidence and enforcing structure-level consistency Alis et al. (2022). In contrast, the proposed method does not rely solely on backbone replacement, multi-slice aggregation, or conventional attention enhancement. Instead, it incorporates counterfactual intervention to suppress lesion-irrelevant but highly correlated features, while introducing domain-invariant consistency regularization to improve both representation robustness and prediction stability under in-domain perturbations. Through this design, the proposed framework establishes a clearer methodological distinction from existing studies and more explicitly highlights its contribution to robust and reliable stroke CT classification.

### 3.2 Overall algorithm architecture

**FIGURE 1:**
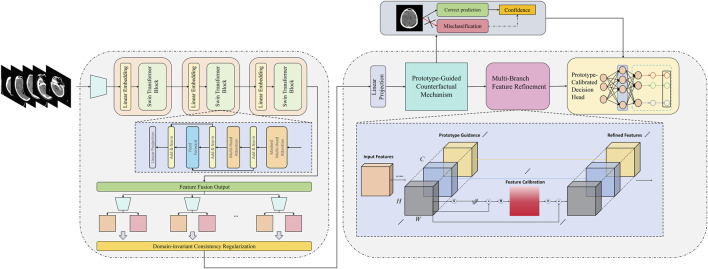
Overall framework of the proposed Swin-Transformer-based head CT stroke classification model. The backbone extracts hierarchical feature representations, while domain-invariant consistency regularization improves prediction stability under in-domain variations such as window width/level perturbations and noise. On the counterfactual side, a prototype-guided counterfactual mechanism and a multi-branch feature refinement module are introduced to enhance lesion-relevant evidence and suppress misleading cues. Finally, a prototype-calibrated decision head produces the class prediction and confidence score in an end-to-end manner.

As illustrated in Figure 1, our method adopts a Swin-Transformer as the backbone to learn discriminative representations for head CT stroke classification,and appends a lightweight counterfactual branch together with prototype-enhanced representation learning and confidence-aware consistency regularization. This design improves classification accuracy and predictive stability while preserving the standard supervised learning setting. Specifically, a single CT slice x is first processed by a patch/linear embedding layer and hierarchical Swin-Transformer stages to extract multi-scale features. The extracted features are then fused into a global representation z , which is fed into a classification head to produce class probabilities. In parallel, a counterfactual mechanism constructs a contrastive representation $z^{\text{cf}}$ based on z to explicitly attenuate spurious factors that are diagnostically irrelevant but easily exploited by the model, thereby encouraging decisions to rely more on stable lesion evidence. The overall forward process can be summarized as shown in Equation 1:

$$z=\Phi_{\text{swin}}\left(x\right),\;z^{\text{cf}}=\Psi_{\text{cf}}\left(z\right),\;\hat{y}=softmax\;\left(g\;\left(\left[z,\;z^{\text{cf}}\right]\right)\right),$$ (1)

where $\Phi_{\text{swin}}\left(\cdot \right)$ denotes the Swin-Transformer backbone with the feature fusion module, $\Psi_{\text{cf}}\left(\cdot \right)$ denotes the counterfactual generation/transformation module, [⋅,⋅] represents feature concatenation, and g(⋅) denotes the discriminative head that will be realized by a prototype-calibrated fusion-and-classification operator in the present framework.

For optimization, we adopt a unified objective that integrates task supervision, counterfactual constraints, and consistency regularization. The task supervision term enforces separability with respect to the ground-truth labels. The counterfactual constraint encourages the original representation and the counterfactual representation to form a stable contrast on lesion-relevant evidence, thereby suppressing the dominance of non-causal cues in the decision process. In addition, the Domain-invariant Consistency Regularization is not intended for domain-adaptive alignment; instead, it applies structure-preserving perturbations to the same slice and constrains prediction consistency, making the model more stable under in-domain variations such as window mapping fluctuations, noise, and mild geometric perturbations, thus improving overall accuracy. The final training objective is formulated as:

$$\underset{\theta }{\min}\;L\left(\theta \right)=L_{\text{cls}}\left(y,\hat{y}\right)+\lambda_{\text{cf}}L_{\text{cf}}\;\left(z,z^{\text{cf}}\right)+\lambda_{\text{con}}L_{\text{con}}\;\left(f_{\theta }\left(A_{1}\left(x\right)\right),f_{\theta }\left(A_{2}\left(x\right)\right)\right),$$ (2)

where θ denotes all learnable parameters, $L_{\text{cls}}$ is the cross-entropy classification loss, $L_{\text{cf}}$ is the constraint term associated with counterfactual learning, and $L_{\text{con}}$ is the consistency regularization term. $A_{1}\left(\cdot \right)$ and $A_{2}\left(\cdot \right)$ denote two structure-preserving perturbations applied to the same input slice (e.g., different window mappings, noise injection, or mild geometric transformations), while $\lambda_{\text{cf}}$ and $\lambda_{\text{con}}$ are weighting coefficients. With this design, the model retains the global modeling capability of Swin-Transformer, while further reducing reliance on spurious correlations via counterfactual contrast and improving prediction stability under in-domain perturbations through consistency constraints, thereby yielding more reliable discriminative performance for head CT stroke classification.

### 3.3 Domain-invariant consistency regularization

**FIGURE 2:**
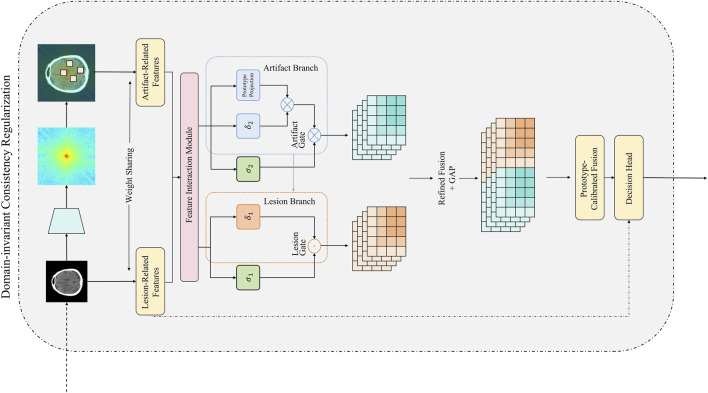
Architecture of the Domain-invariant Consistency Regularization (DICR) module. Two anatomy-preserving views are encoded into content-related and domain-related features, which are separately modulated by dual-gated fusion and aggregated into a shared representation for stable classification.

To improve the stability of single-slice (PNG) head CT stroke classification under window width/level variations, noise perturbations, and imaging-style fluctuations, we propose Domain-invariant Consistency Regularization (DICR). The goal of DICR is to enforce consistent discriminative representations and output distributions for the same image under different in-domain views, without introducing domain adaptation or relying on additional domain labels. Its modular architecture is shown in Figure 2.

Let a training sample be $\left(x_{i},y_{i}\right)$ , where $x_{i}\in R^{H\times W\times 1}$ is a single-channel CT slice (PNG) and $y_{i}\in \left\{1,\ldots ,K\right\}$ is the class label; H,W denote the image height and width, and K is the number of classes. Given two anatomy-preserving random augmentation operators $A_{1}\left(\cdot \right)$ and $A_{2}\left(\cdot \right)$ , we construct two views of the same sample as:

$$x_{i}^{\left(1\right)}=A_{1}\left(x_{i}\right),\;x_{i}^{\left(2\right)}=A_{2}\left(x_{i}\right),$$ (3)

where superscripts (1) and (2) denote different views. $A_{1}$ and $A_{2}$ can be instantiated as different window mappings, strong/weak brightness–contrast perturbations, mild blur/noise injection, or slight geometric perturbations, which simulate in-domain imaging differences without changing the lesion semantic category. Equation 3 explicitly converts “in-domain uncertainty” into multi-view representations of the same input, providing the basis for subsequent consistency modeling.

At the representation level, we extract both content-related features and domain-related features, and require them to be controllably fused within a shared space. Let the encoder be $E_{\theta }\left(\cdot \right)$ with parameters θ , whose output feature dimensionality is $C\times H^{\prime }\times W^{\prime }$ . The view-specific content features are defined as:

$$F_{i}^{\left(v\right)}=E_{\theta }\;\left(x_{i}^{\left(v\right)}\right),\;v\in \left\{1,2\right\},$$ (4)

where $F_{i}^{\left(v\right)}\in R^{C\times H^{\prime }\times W^{\prime }}$ , C is the channel number, $H^{\prime },W^{\prime }$ are the spatial dimensions of feature maps, and v is the view index. To explicitly represent “domain information”, we construct a domain transformation T(⋅) for each view (e.g., frequency-domain amplitude spectrum mapping, style-statistics mapping, or a normalized texture view), and use the same encoder to extract domain features:

$${\tilde{x}}_{i}^{\left(v\right)}=T\;\left(x_{i}^{\left(v\right)}\right),\;D_{i}^{\left(v\right)}=E_{\theta }\;\left({\tilde{x}}_{i}^{\left(v\right)}\right),$$ (5)

where ${\tilde{x}}_{i}^{\left(v\right)}$ is the domain view and $D_{i}^{\left(v\right)}\in R^{C\times H^{\prime }\times W^{\prime }}$ is the domain feature. Equations 4, 5 encode “content evidence related to lesions” and “domain cues related to imaging style” in the same feature space, enabling learnable modeling of domain factors without explicit domain labels. To reduce training instability caused by scale mismatch, we normalize the features as:

$${\bar{F}}_{i}^{\left(v\right)}=Norm\;\left(F_{i}^{\left(v\right)}\right),\;{\bar{D}}_{i}^{\left(v\right)}=Norm\;\left(D_{i}^{\left(v\right)}\right),$$ (6)

where Norm(⋅) performs $\ell_{2}$ normalization on the channel vector at each spatial location, so that different views and branches have comparable magnitude ranges. ${\bar{F}}_{i}^{\left(v\right)}$ and ${\bar{D}}_{i}^{\left(v\right)}$ remain in $R^{C\times H^{\prime }\times W^{\prime }}$ .

For fusion and gating, we employ dot-product interaction to capture co-activated regions between content evidence and domain cues, and apply a dual-gating scheme to modulate the features with the principle of retaining stable evidence and suppressing unstable responses. This design is based on the observation that content features and domain features play fundamentally different roles in stroke CT recognition. Content features should retain lesion-related discriminative information as much as possible, whereas domain features require more careful regulation because they may encode variations related to window settings, noise patterns, and artifact-induced fluctuations. Because lesion-related content features should be preserved while domain-related responses need to be selectively controlled, a directly fused or single-gate strategy would be less suitable, as it may either over-suppress discriminative lesion evidence or over-retain unstable contextual cues. Therefore, rather than adopting direct fusion or a single shared gate, we employ dual-gated modulation to regulate the two branches separately, so that the final representation can achieve better stability and interpretability. We first define the dot-product fused feature as:

$$U_{i}^{\left(v\right)}={\bar{F}}_{i}^{\left(v\right)}⊙{\bar{D}}_{i}^{\left(v\right)},$$ (7)

where ⊙ denotes element-wise multiplication and $U_{i}^{\left(v\right)}\in R^{C\times H^{\prime }\times W^{\prime }}$ . Equation 7 highlights positions/channels where both content and domain features are strongly activated, providing a relevance hint for subsequent gating. We then construct the first gating branch to modulate the content feature, with the gating weight defined as:

$$g_{1,i}^{\left(v\right)}=\sigma \;\left(W_{1}*U_{i}^{\left(v\right)}+b_{1}\right),$$ (8)

where $W_{1}$ is a learnable convolution kernel (or an equivalent linear mapping), ∗ denotes convolution, $b_{1}$ is the bias, and σ(⋅) is the sigmoid function that squashes outputs into [0,1]. Thus, $g_{1,i}^{\left(v\right)}\in {\left[0,1\right]}^{C\times H^{\prime }\times W^{\prime }}$ represents the proportion of retaining the original content evidence. The modulated content feature is then given by:

$${\hat{F}}_{i}^{\left(v\right)}=g_{1,i}^{\left(v\right)}⊙F_{i}^{\left(v\right)}+\left(1-g_{1,i}^{\left(v\right)}\right)⊙\left(W_{2}*U_{i}^{\left(v\right)}+b_{2}\right),$$ (9)

where 1 is an all-ones tensor with the same shape as $g_{1,i}^{\left(v\right)}$ , and $W_{2},b_{2}$ are another set of learnable parameters; ${\hat{F}}_{i}^{\left(v\right)}\in R^{C\times H^{\prime }\times W^{\prime }}$ . Equation 9 implies that when $g_{1,i}^{\left(v\right)}$ is large, stable lesion evidence in $F_{i}^{\left(v\right)}$ is largely preserved; when $g_{1,i}^{\left(v\right)}$ is small, an alternative expression generated from $U_{i}^{\left(v\right)}$ compensates for responses that may be corrupted by perturbations, thereby suppressing unstable content features. This branch is intended to adaptively preserve lesion-related information during fusion, so as to prevent clinically meaningful evidence from being excessively contaminated by unstable responses. The second gating branch modulates the domain feature, with the gating weight and linear projection defined as:

$$g_{2,i}^{\left(v\right)}=\sigma \;\left(W_{3}*U_{i}^{\left(v\right)}+b_{3}\right),\;L_{i}^{\left(v\right)}=W_{4}*U_{i}^{\left(v\right)}+b_{4},$$ (10)

where $W_{3},W_{4}$ and $b_{3},b_{4}$ are learnable parameters, $g_{2,i}^{\left(v\right)}\in {\left[0,1\right]}^{C\times H^{\prime }\times W^{\prime }}$ , and $L_{i}^{\left(v\right)}\in R^{C\times H^{\prime }\times W^{\prime }}$ . Accordingly, the modulated domain feature is:

$${\hat{D}}_{i}^{\left(v\right)}=g_{2,i}^{\left(v\right)}⊙D_{i}^{\left(v\right)}+\left(1-g_{2,i}^{\left(v\right)}\right)⊙L_{i}^{\left(v\right)},$$ (11)

where ${\hat{D}}_{i}^{\left(v\right)}\in R^{C\times H^{\prime }\times W^{\prime }}$ . Equations 10, 11 constrain the domain feature to be smoother and more controllable while retaining necessary style statistics, preventing the model from over-exploiting incidental fluctuations in domain textures. In contrast to the first gate, the second branch is primarily introduced to suppress unstable style-related responses, allowing domain cues to serve as auxiliary contextual information without exerting undue influence on the final decision. Finally, we aggregate the two modulated branches into a shared representation vector:

$$z_{i}^{\left(v\right)}=Concat\;\left(GAP\;\left({\hat{F}}_{i}^{\left(v\right)}\right),GAP\;\left({\hat{D}}_{i}^{\left(v\right)}\right)\right),$$ (12)

where GAP(⋅) denotes global average pooling that compresses $C\times H^{\prime }\times W^{\prime }$ into a vector in $R^{C}$ , and Concat(⋅,⋅) denotes vector concatenation; hence $z_{i}^{\left(v\right)}\in R^{2C}$ . Equation 12 combines gated content evidence and gated, smoothed domain cues into the final discriminative representation, providing a more stable and robust input to the classifier.

At the output level, we obtain class probabilities via a linear mapping and softmax, and define consistency measures at both the probability and representation levels (note that only the mathematical forms of the consistency constraints are given here, without expanding any optimization objective or loss function). Let the classification head be $h_{\psi }\left(\cdot \right)$ with parameters ψ . The predicted probabilities for the two views are:

$$p_{i}^{\left(v\right)}=softmax\;\left(h_{\psi }\;\left(z_{i}^{\left(v\right)}\right)\right),\;p_{i}^{\left(v\right)}\in \Delta^{K},$$ (13)

where $\Delta^{K}$ denotes the K -dimensional probability simplex (each component is non-negative and sums to 1), and the k -th component $p_{i,k}^{\left(v\right)}$ denotes the probability of predicting class k under view v . To quantify the agreement between the two predictive distributions, we use the symmetric KL divergence:

$$D_{\text{sym}}\;\left(p_{i}^{\left(1\right)},p_{i}^{\left(2\right)}\right)=\frac{1}{2}\left(KL\left(p_{i}^{\left(1\right)}\|p_{i}^{\left(2\right)}\right)+KL\left(p_{i}^{\left(2\right)}\|p_{i}^{\left(1\right)}\right)\right),$$ (14)

where $KL\left(a\|b\right)=\sum_{k=1}^{K}a_{k}\log\frac{a_{k}}{b_{k}}$ is the KL divergence; a smaller $D_{\text{sym}}\left(\cdot ,\cdot \right)$ indicates more consistent output distributions across views. Meanwhile, to measure representation-level stability, we apply the squared Euclidean distance on $\ell_{2}$ -normalized shared representations:

$$D_{z}\;\left(z_{i}^{\left(1\right)},z_{i}^{\left(2\right)}\right)={\left\|\frac{z_{i}^{\left(1\right)}}{\|z_{i}^{\left(1\right)}{\|}_{2}}-\frac{z_{i}^{\left(2\right)}}{\|z_{i}^{\left(2\right)}{\|}_{2}}\right\|}_{2}^{2},$$ (15)

where $\|\cdot {\|}_{2}$ is the $\ell_{2}$ norm; normalization removes the influence of magnitude differences on the distance, and a smaller $D_{z}\left(\cdot ,\cdot \right)$ indicates more stable representations across views. In summary, DICR explicitly separates and fuses content evidence and domain cues via gated interactions as in Equations 3–12, and characterizes consistency at both the output and representation levels via Equations 13–15, thereby enhancing robustness to in-domain perturbations and improving predictive trustworthiness without introducing domain adaptation.

Different from the original uniform consistency formulation, the present framework further introduces a prototype-calibrated reliability coefficient to modulate the consistency strength. The view-specific reliability score and the averaged reliability coefficient are defined in Equation 16, and the final weighted consistency term is implemented in Equation 17. Let $M=\left\{\mu_{1},\mu_{2},\ldots ,\mu_{K}\right\}$ denote the learnable prototype set that will also be used in the counterfactual branch. For each view-specific representation $z_{i}^{\left(v\right)}$ , we define its reliability by the distance to the nearest class prototype as:

$$c_{i}^{\left(v\right)}=\exp\left(-\gamma \underset{k}{\min}\|z_{i}^{\left(v\right)}-\mu_{k}{\|}_{2}\right),\;{\bar{c}}_{i}=\frac{c_{i}^{\left(1\right)}+c_{i}^{\left(2\right)}}{2}$$ (16)

where γ>0 is a scaling factor, $c_{i}^{\left(v\right)}\in \left(0,1\right]$ denotes the reliability of the v -th view, and ${\bar{c}}_{i}$ denotes the average reliability of the two views. A representation located far away from all class prototypes therefore receives a lower reliability score, even if its relative class distribution appears sharp. Accordingly, the consistency term in Equation 2 is implemented as:

$$L_{\text{con}}=\frac{1}{N}\overset{N}{\underset{i=1}{\sum }}{\bar{c}}_{i}\left[\lambda_{p}D_{\text{sym}}\left(p_{i}^{\left(1\right)},p_{i}^{\left(2\right)}\right)+\lambda_{z}D_{z}\left(z_{i}^{\left(1\right)},z_{i}^{\left(2\right)}\right)\right]$$ (17)

where $\lambda_{p}>0$ and $\lambda_{z}>0$ denote the weighting coefficients of the probability-level and representation-level consistency terms, respectively. In this way, reliable samples receive stronger consistency supervision, whereas highly uncertain samples are prevented from imposing misleading regularization. This design preserves the original DICR structure while explicitly absorbing the core idea of uncertainty-aware consistency regularization.

### 3.4 Counterfactual mechanism

**FIGURE 3:**
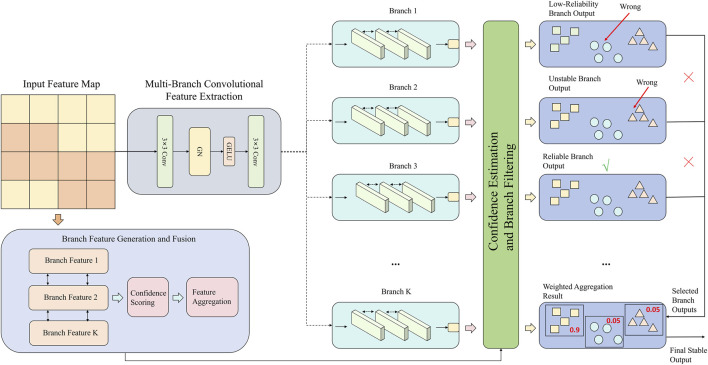
The figure illustrates the confidence estimation process based on multi-branch convolutional feature extraction and fusion. By evaluating the predictions of individual branches, the model filters out unreliable or unstable branch outputs, suppresses erroneous judgments, and then weights and aggregates the retained reliable branches to obtain the final stable output.

In the domain-invariant consistency regularization of the previous subsection, we explicitly reduced instabilities caused by window mapping, noise, and mild style fluctuations by applying structure-preserving perturbations to the same PNG slice and enforcing representation/prediction consistency; however, consistency alone may still encourage the model to stably exploit some diagnostically irrelevant yet highly correlated spurious cues in the training set. To further mitigate the influence of such spurious correlations, we design a Counterfactual mechanism, whose core idea is to construct a counterfactual representation that simulates how the model should discriminate when non-causal factors are intervened upon, while keeping the primary anatomical structure of the input slice unchanged, and to use this counterfactual representation together with the original representation for final classification. The experimental results are shown in Figure 3.

Let the input slice be $x\in R^{H\times W\times 1}$ , and let the encoder (the Swin-Transformer backbone with a fusion layer) be $\Phi_{\text{swin}}\left(\cdot \right)$ . The original global representation is defined as:

$$z=\Phi_{\text{swin}}\left(x\right)\in R^{d},$$ (18)

where H,W denote the image resolution and d is the representation dimension. Equation 18 maps a single CT slice into a compact vector space for discrimination, serving as the common starting point for counterfactual construction and final prediction.

Before decomposing z into causal and confounding parts, we explicitly introduce a Learnable Prototype-Enhanced Module (LPEM) so that prototype learning becomes a core component of the present framework rather than merely an auxiliary calibration tool. Let $M=\left\{\mu_{1},\mu_{2},\ldots ,\mu_{K}\right\}$ denote the learnable prototype set, where $\mu_{k}\in R^{d}$ is the prototype corresponding to class k . We first compute the cosine similarity between z and each class prototype as shown in Equation 19.

$$sim\left(z,\mu_{k}\right)=\frac{z^{⊤}\mu_{k}}{|z{|}_{2}|\mu_{k}{|}_{2}}$$ (19)

where $sim\left(z,\mu_{k}\right)\in \left[-1,1\right]$ measures the directional similarity between the sample feature and the k -th class anchor. To encourage z to be closer to the prototype of its ground-truth class y and farther away from prototypes of other classes, we define a prototype contrastive loss as shown in Equation 20.

$$L_{\text{proto}}=-\log\frac{\exp\left(sim\left(z,\mu_{y}\right)/\tau_{\text{proto}}\right)}{\sum_{k=1}^{K}\exp\left(sim\left(z,\mu_{k}\right)/\tau_{\text{proto}}\right)}$$ (20)

where $\tau_{\text{proto}}>0$ denotes the temperature parameter controlling the sharpness of the similarity distribution. To utilize the learned prototypes for feature enhancement, we compute prototype attention weights and construct a residual prototype-enhanced feature as shown in Equation 21.

$$a_{k}=\frac{\exp\left(sim\left(z,\mu_{k}\right)/\tau_{\text{fuse}}\right)}{\sum_{j=1}^{K}\exp\left(sim\left(z,\mu_{j}\right)/\tau_{\text{fuse}}\right)},\;z^{e}=z+\beta \overset{K}{\underset{k=1}{\sum }}a_{k}\mu_{k}$$ (21)

where $a_{k}\in \left(0,1\right)$ denotes the contribution of the k -th prototype to the current sample, $\tau_{\text{fuse}}>0$ denotes the fusion temperature, and β denotes a learnable scaling factor. Equation 21 injects class-structured information into the original feature while preserving the sample-specific visual evidence through a residual pathway. To ensure that the prototypes stably represent global class centers, we update them by exponential moving average as shown in Equation 22.

$${\bar{z}}_{k}=\frac{1}{\left|B_{k}\right|}\underset{z_{i}\in B_{k}}{\sum }z_{i},\;\mu_{k}\leftarrow m_{p}\mu_{k}+\left(1-m_{p}\right){\bar{z}}_{k}$$ (22)

where $\left|B_{k}\right|$ denotes the number of class- k samples in the current mini-batch, and $m_{p}\in \left[0,1\right)$ denotes the prototype momentum coefficient. In the remainder of this subsection, for notational simplicity and to preserve the original equation chain as much as possible, the prototype-enhanced representation $z_{e}$ is still denoted by z .

To formalize interventions on “spurious factors,” we decompose z into two components: a causal component relevant to lesion discrimination and a confounding component related to imaging style/artifacts. Specifically, we introduce two learnable projection operators $P_{c}\in R^{d\times d}$ and $P_{s}\in R^{d\times d}$ , and define:

$$z_{c}=P_{c}z,\;z_{s}=P_{s}z,$$ (23)

where $z_{c}\in R^{d}$ denotes the “causal/lesion-related” sub-representation and $z_{s}\in R^{d}$ denotes the “confounding/spurious” sub-representation. In the present framework, $P_{c}$ and $P_{s}$ are implemented in a prototype-guided manner, so that $z_{c}$ is biased toward class-consistent lesion evidence while $z_{s}$ captures prototype-deviation residuals more closely associated with artifacts and other spurious cues.

To avoid degenerate decompositions caused by arbitrary overlap between the two components, we further provide an algebraic form of an orthogonalization constraint at the representation level (only the structural definition is given here, without introducing any loss term). We first normalize the two sub-representations as:

$${\bar{z}}_{c}=\frac{z_{c}}{\|z_{c}{\|}_{2}},\;{\bar{z}}_{s}=\frac{z_{s}}{\|z_{s}{\|}_{2}},$$ (24)

where $\|\cdot {\|}_{2}$ is the $\ell_{2}$ norm and ${\bar{z}}_{c},{\bar{z}}_{s}\in R^{d}$ are unit vectors. We then measure their correlation via an inner product:

$$\rho =\left\langle {\bar{z}}_{c},{\bar{z}}_{s}\right\rangle =\overset{d}{\underset{j=1}{\sum }}{\bar{z}}_{c,j}{\bar{z}}_{s,j},$$ (25)

where ${\bar{z}}_{c,j}$ and ${\bar{z}}_{s,j}$ are the j -th components and ρ∈[−1,1] , with values closer to 0 indicating better decoupling between the subspaces. Equations 23–25 decompose the original representation into two controllable parts and provide a computable measure of “decoupling degree,” enabling counterfactual intervention to act explicitly on $z_{s}$ while minimizing disruption to $z_{c}$ .

Based on this decomposition, we construct the counterfactual representation by intervening on the confounding component. We first define a generic intervention operator I(⋅) as shown in Equation 26, that performs suppression, resampling, or replacement on $z_{s}$ to produce a counterfactual confounding component:

$$z_{s}^{\text{cf}}=I\left(z_{s}\right).$$ (26)

Here $z_{s}^{\text{cf}}\in R^{d}$ denotes the “intervened” confounding representation. To make I(⋅) both learnable and interpretable with controllable behavior, we adopt a gated intervention form. We first estimate a confounder-suppression gate, as shown in Equation 27:

$$m=\sigma \left(W_{m}z+b_{m}\right)\in {\left(0,1\right)}^{d},$$ (27)

where $W_{m}\in R^{d\times d}$ is a learnable matrix, $b_{m}\in R^{d}$ is a bias vector, and σ(⋅) is the sigmoid function applied element-wise. Each component $m_{j}$ indicates the retention ratio of confounding information in the j -th dimension.

We then introduce a reference confounding prototype $r\in R^{d}$ as a replacement basis and define the intervened confounding component as:

$$z_{s}^{cf}=m⊙z_{s}+\left(1-m\right)⊙r$$ (28)

where ⊙ denotes element-wise multiplication. In contrast to an unconstrained reference vector, r is constructed from non-dominant class prototypes, so that the replacement operation remains tied to the learned class structure. Equation 28 imply that when certain dimensions are deemed more likely to carry spurious correlations, the gate m reduces their inherited contribution from $z_{s}$ and replaces them with a more neutral prototype-derived reference r , thereby simulating a counterfactual scenario in which confounding factors are altered while the model should still maintain stable lesion discrimination.

The final counterfactual global representation is then composed by combining the causal component with the counterfactually intervened confounding component:

$$z^{cf}=\Psi_{\text{cf}}\left(z\right)=\alpha z_{c}+\left(1-\alpha \right)z_{s}^{cf}$$ (29)

where α∈[0,1] is a mixing coefficient, which can be set as a constant or predicted adaptively by a small network. Equation 29 constructs a contrastive representation by preserving lesion-related information while injecting the intervened confounding information, thereby revealing the model’s discriminative tendency after suppressing prototype-inconsistent spurious cues.

At inference and fusion time, we feed the original representation and the counterfactual representation jointly into a discriminative head to achieve unified decision-making with evidence complementarity and spurious-correlation suppression. Let the fused feature be defined as in Equation 30:

$$h=\Gamma \left(\left[z,z^{cf}\right]\right)=\phi \left(W_{h}\left[z,z^{cf}\right]+b_{h}\right)\in R^{d_{h}}$$ (30)

where $\left[z,z^{cf}\right]\in R^{2d}$ denotes vector concatenation, $W_{h}\in R^{d_{h}\times 2d}$ and $b_{h}\in R^{d_{h}}$ are learnable parameters, ϕ(⋅) is a nonlinear activation function, and $d_{h}$ is the fusion feature dimension. In the present framework, Γ(⋅) includes the multi-branch feature refinement strategy of the Prototype-Calibrated Confidence Mechanism (PCCM), so that the fused feature h serves as the refined decision feature before confidence calibration. This explicitly preserves the value of prototype-calibrated decision rather than leaving it as an implicit implementation detail.

To further improve decision reliability, we introduce prototype-calibrated confidence estimation on the fused feature h . Let ${\bar{\mu }}_{k}$ denote the projected prototype of class k in the fusion space. We compute the Euclidean distance between *h* and each projected prototype and obtain a prototype-induced class distribution as shown in Equation 31.

$$d\left(h,{\bar{\mu }}_{k}\right)=|h-{\bar{\mu }}_{k}{|}_{2},\;{\tilde{p}}_{k}=\frac{\exp\left(-d\left(h,{\bar{\mu }}_{k}\right)\right)}{\sum_{j}\exp\left(-d\left(h,{\bar{\mu }}_{j}\right)\right)}$$ (31)

where $d\left(h,{\bar{\mu }}_{k}\right)$ denotes the distance to the k -th class prototype in the fusion space, and ${\tilde{p}}_{k}$ denotes the corresponding prototype-induced class probability. The initial confidence score is then defined in Equation 32 as:

$$c_{\text{init}}=\underset{k}{\max}{\tilde{p}}_{k}$$ (32)

To calibrate this confidence, we introduce a prototype-based calibration signal, defined in Equation 33, that is inversely proportional to the distance to the nearest prototype.

$$s_{\text{cal}}=\exp\left(-\gamma \underset{k}{\min}d\left(h,{\bar{\mu }}_{k}\right)\right)$$ (33)

where γ>0 is a scaling hyper-parameter. A fused feature located far away from all known prototypes therefore receives a lower calibration score, even if its relative class distribution appears sharp. A confidence gate is then learned to dynamically fuse the initial confidence and the calibration signal:

$$g_{c}=\sigma \left(W_{g}\left[c_{\text{init}},s_{\text{cal}}\right]+b_{g}\right),\;\tilde{h}=g_{c}\cdot h$$ (34)

where $W_{g}$ and $b_{g}$ are learnable parameters, and $\left[c_{\text{init}},s_{\text{cal}}\right]$ denotes scalar concatenation.

The output logits and probability distribution are then given by:

$$s=W_{o}\tilde{h}+b_{o}\in R^{K},\;\hat{y}=softmax\left(s\right)\in \Delta^{K}$$ (35)

where $W_{o}\in R^{K\times d_{h}}$ and $b_{o}\in R^{K}$ denote the output-layer parameters. Compared with a plain fusion-to-logits mapping, Equations 34, 35 explicitly incorporate prototype-distance calibration into the final decision stage, so that features lying far away from all class anchors are less likely to produce unreliable overconfident predictions. This is exactly the core role of prototype-calibrated confidence in the original PCCM design.

To further characterize how the counterfactual branch modulates model confidence, we define the predictive confidence by the maximum class probability, as shown in Equation 36.

$$conf\left(x\right)=\underset{k\in \left\{1,\ldots ,K\right\}}{\max}{\hat{y}}_{k}$$ (36)

where ${\hat{y}}_{k}$ denotes the probability of class k . A larger conf(x) indicates that the model is more certain about a particular class. In the present framework, the counterfactual branch guides the model to allocate high confidence more often to predictions supported by stable lesion evidence, while the prototype-distance calibration suppresses overconfident decisions for fused features located far away from all class anchors.

Together with the confidence-aware consistency regularization described in Section 3.3, the overall framework improves not only the stability of “the same image under different views” but also the reliability of “which evidence the model stably relies on” during head CT stroke classification.

## 4 Datasets and evaluation metrics

### 4.1 Datasets

**FIGURE 4:**
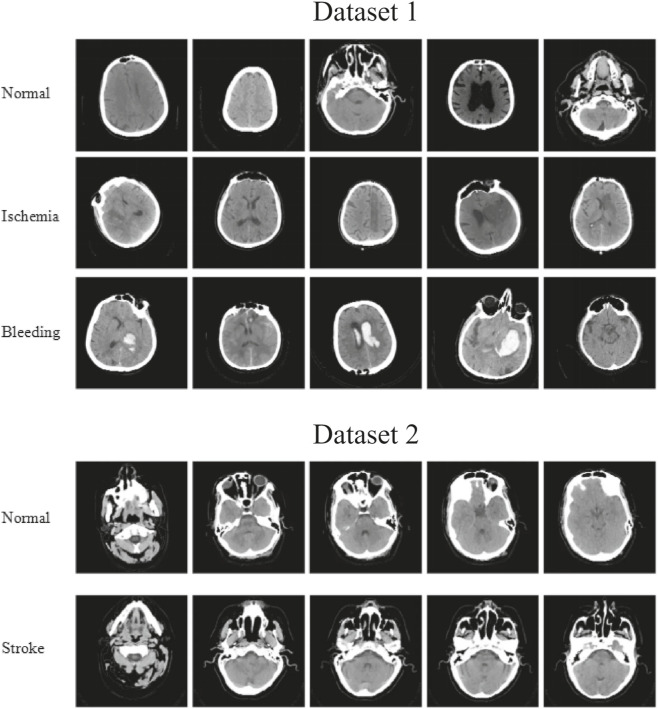
Example images from Dataset1 and Dataset2.

This paper conducts experiments on two head CT stroke classification datasets. The first dataset was curated and released under the support of the Turkish Ministry of Health for the Artificial Intelligence in Healthcare competition held in Istanbul in 2021. It contains 6,653 brain CT slice images and is divided into three classes: No stroke (4,428 images), Ischemia (1,131 images), and Bleeding (1,094 images). The second dataset is sourced from Kaggle and formulates a binary classification task with 950 Normal images and 1,551 Stroke images, totaling 2,501 images. To ensure fair and reproducible evaluation, both datasets are split into training, validation, and test sets using stratified splitting with a ratio of 8:1:1, such that the class proportions in each subset remain as consistent as possible with the original distribution. Detailed statistics are reported in Table 2. Furthermore, example images from two datasets are provided, as shown in Figure 4.

**TABLE 2:** Class distribution and train/validation/test split statistics for the two datasets (stratified 8:1:1).

Dataset	Class	Total	Train	Val	Test
AI in Healthcare 2021	No stroke	4,428	3,542	443	443
	Ischemia	1,131	905	113	113
	Bleeding	1,094	875	110	109
	Total	6,653	5,322	666	665
Kaggle Stroke/Normal	Normal	950	760	95	95
	Stroke	1,551	1,240	156	155
	Total	2,501	2000	251	250

### 4.2 Evaluation metrics

Accuracy (Acc) measures the proportion of correctly predicted samples and provides an intuitive reflection of overall classification performance. Let TP , TN , FP , and FN denote true positives, true negatives, false positives, and false negatives, respectively. Accuracy is defined in Equation 37 as:

$$Acc=\frac{TP+TN}{TP+TN+FP+FN}.$$ (37)

The denominator is the total number of samples, and the numerator is the number of correctly classified samples.

Precision measures the proportion of truly positive samples among all samples predicted as positive, reflecting the ability to control false positives. Precision is defined in Equation 38 as:

$$Precision=\frac{TP}{TP+FP}.$$ (38)

A higher precision indicates that positive predictions are more reliable and that there are fewer false alarms.

Recall measures the proportion of truly positive samples that are successfully detected by the model, reflecting the ability to control false negatives. Recall is defined in Equation 39 as:

$$Recall=\frac{TP}{TP+FN}.$$ (39)

A higher recall indicates better coverage of positive samples and fewer missed detections.

The F1-score is the harmonic mean of Precision and Recall, and it is used to balance false positives and false negatives. The F1-score is defined in Equation 40 as:

$$F1=\frac{2\cdot Precision\cdot Recall}{Precision+Recall}.$$ (40)

The F1-score becomes large only when both Precision and Recall are high.

The Area Under the ROC Curve (AUC) measures the overall ability of a model to distinguish positive from negative samples across different decision thresholds, independent of a fixed threshold. AUC is defined in Equation 41 as the integral area under the ROC curve:

$$AUC=\overset{1}{\underset{0}{\int }}TPR\left(t\right)\;d\;FPR\left(t\right).$$ (41)

Here, TPR(t) and FPR(t) denote the true positive rate and false positive rate at threshold t , respectively.

## 5 Experiment

### 5.1 Experimental setup

This study adopts a unified training pipeline on both datasets to ensure fair comparison. All CT slices are provided to the model in PNG format. During training, images are resized and normalized, and random data augmentation is applied to improve robustness. The model uses Swin-Transformer as the backbone and is trained end-to-end. The validation set is used for hyper-parameter selection and early stopping, and all evaluation metrics are finally reported on the test set. The key hyper-parameter settings are summarized in Table 3.

**TABLE 3:** Hyper-parameter settings.

Hyper-parameter	Value
Input image size	224×224
Backbone	Swin-Transformer
Optimizer	AdamW
Initial learning rate	$1\times 10^{-4}$
Weight decay	$5\times 10^{-2}$
Batch size	32
Training epochs	200
LR scheduler	Cosine Annealing
Warmup epochs	5
Dropout	0.1
Label smoothing	0.0
Gradient clipping (max norm)	1.0
Random seed	2025
Temperature $\left(\tau_{\text{proto}}\right)$	0.1
Temperature $\left(\tau_{\text{fuse}}\right)$	1.0
Calibration scaling (γ)	2.0
Prototype EMA momentum $\left(m_{p}\right)$	0.99

The overall optimization follows the joint objective of classification supervision, consistency regularization, and counterfactual constraint. Prototype-enhanced representation learning and prototype-calibrated decision are embedded into the counterfactual branch, while confidence-aware reliability weighting is incorporated into the consistency branch. Therefore, the training procedure introduces more structured feature organization and more reliable confidence modulation within a unified optimization framework.

### 5.2 Experimental results compared with other models

To comprehensively evaluate the effectiveness and substitutability of the proposed method for head CT slice-based stroke classification, we conduct comparative experiments under the same data split, preprocessing, and training strategy, covering classical convolutional networks, modern convolutional backbones, and Transformer-based architectures. Specifically, the baseline models include ResNet He et al. (2016), ResNeXt Xie et al. (2017), ConvNeXtV2 Woo et al. (2023), and Vision Transformer Dosovitskiy (2020). We also include stroke-oriented methods, namely StrokeNeXt Ekingen et al. (2025), Stro-VIGRU Das et al. (2025), StrokeVIT Raj et al. (2023), and GACL-Net Bunterngchit et al. (2025), to ensure representative and sufficient comparisons. All methods are evaluated using the same metrics and testing protocol, enabling an objective analysis of performance differences across architectural paradigms in the stroke classification task and validating the overall advantages of our approach. First, we present the experimental results for dataset 1, which are shown in Table 4.

**TABLE 4:** Comparative results with representative baselines (mean ± std). The best performance is highlighted (dataset1).

Method	Acc	Precision	Recall	F1-score	AUC	FPS
ResNet	0.8998±0.0111	0.8914±0.0122	0.8725±0.0149	0.8817±0.0119	0.9506±0.0090	137.9
ResNeXt	0.9187±0.0096	0.9057±0.0104	0.8893±0.0135	0.8996±0.0103	0.9583±0.0076	121.7
ConvNeXtV2	0.9354±0.0086	0.9280±0.0093	0.9074±0.0117	0.9154±0.0089	0.9669±0.0056	98.3
VIT	0.9258±0.0094	0.9150±0.0113	0.8974±0.0119	0.9066±0.0094	0.9666±0.0068	62.9
StrokeNeXt	0.9500±0.0067	0.9409±0.0077	0.9234±0.0094	0.9302±0.0067	0.9777±0.0047	113.5
Stro-VIGRU	0.9427±0.0075	0.9327±0.0081	0.9170±0.0101	0.9279±0.0076	0.9777±0.0052	86.7
StrokeVIT	0.9508±0.0065	0.9444±0.0082	0.9300±0.0094	0.9390±0.0058	0.9815±0.0043	71.3
GACL-Net	0.9585±0.0058	0.9520±0.0069	0.9372±0.0079	0.9419±0.0060	0.9842±0.0034	104.1
Ours	0.9823±0.0034	0.9766±0.0026	0.9740±0.0025	0.9805±0.0030	0.9982±0.0009	92.7

From the overall results, the proposed method achieves the best performance on all five core discrimination metrics. Acc, Recall, F1, and AUC reach 0.9823, 0.9740, 0.9805, and 0.9982, respectively. Compared with the strongest competing methods (e.g., GACL-Net and StrokeVIT), it still shows consistent improvements, indicating that the framework not only increases overall correctness but also significantly strengthens positive-sample coverage and threshold-independent discriminative ability. Considering the architectural design, these consistent gains align with the multi-scale modeling advantage brought by the hierarchical window-based attention of Swin-Transformer, which can capture both local texture variations and global structural differences, providing a stronger representational foundation for fine-grained stroke-related lesion cues. On top of this, the convolutional enhancement branch further compensates for the limited sensitivity of Transformers to local edges and subtle density gradients, making the model less likely to be distracted by local noise or background artifacts when distinguishing confusing classes such as ischemia and bleeding, which is reflected by the simultaneous increase of Accuracy, Recall, F1, and AUC.

A closer look at the relationship between Precision and Recall shows that the proposed method maintains a high Precision of 0.9766 while achieving a higher Recall of 0.9740, suggesting that it reduces missed detections without substantially increasing false alarms. This balance is directly related to the two key mechanisms introduced in this work. On the one hand, the in-domain consistency regularization applies structure-preserving variations such as window mapping shifts and noise perturbations to the same slice and enforces representation/prediction consistency, which reduces sensitivity to incidental imaging conditions and leads to a smoother and more stable decision boundary. On the other hand, the counterfactual mechanism explicitly suppresses diagnostically irrelevant but easily exploitable spurious factors via contrastive representations, guiding the model to rely more on stable lesion evidence for classification and thereby improving discriminability and the reliability of confidence outputs under perturbations. Meanwhile, the inference speed reaches 92.7 FPS. Although it is lower than lightweight CNNs (e.g., ResNet), it still maintains high efficiency, indicating a relatively reasonable trade-off between accuracy and speed, and making the method more suitable for deployment as a high-accuracy assistant model in clinical screening and large-scale reading scenarios. Further experimental results for dataset 2 are presented in Table 5.

**TABLE 5:** Comparative results with representative baselines (mean ± std). The best performance is highlighted (dataset2).

Method	Acc	Precision	Recall	F1-score	AUC	FPS
ResNet	0.8715±0.0103	0.8600±0.0119	0.8333±0.0147	0.8454±0.0121	0.9368±0.0086	137.9
ResNeXt	0.8882±0.0090	0.8793±0.0099	0.8532±0.0136	0.8642±0.0103	0.9476±0.0075	121.7
ConvNeXtV2	0.9092±0.0086	0.8952±0.0093	0.8725±0.0113	0.8861±0.0090	0.9606±0.0062	98.3
VIT	0.8976±0.0088	0.8861±0.0114	0.8634±0.0117	0.8763±0.0092	0.9536±0.0070	62.9
StrokeNeXt	0.9240±0.0065	0.9159±0.0069	0.9035±0.0090	0.9089±0.0065	0.9739±0.0045	113.5
Stro-VIGRU	0.9197±0.0069	0.9102±0.0083	0.8968±0.0099	0.9004±0.0077	0.9694±0.0054	86.7
StrokeVIT	0.9297±0.0061	0.9241±0.0080	0.9088±0.0090	0.9177±0.0056	0.9778±0.0044	71.3
GACL-Net	0.9388±0.0056	0.9349±0.0071	0.9210±0.0082	0.9265±0.0056	0.9868±0.0038	104.1
Ours	0.9652±0.0033	0.9688±0.0026	0.9773±0.0025	0.9698±0.0030	0.9989±0.0009	92.7

On the binary classification dataset (dataset2), the proposed method also demonstrates stable and significant overall advantages. Acc, F1, and AUC reach 0.9652, 0.9698, and 0.9989, respectively, and it still achieves clear improvements over the strongest baseline GACL-Net (Acc 0.9388, F1 0.9265, AUC 0.9868). In particular, Precision and Recall are both maintained at high levels (0.9688/0.9773), indicating that the model further reduces missed detections while controlling false alarms, which reflects a more stable decision boundary and a stronger focus on critical stroke-related evidence. These results are consistent with the complementary mechanisms of the proposed framework. On the one hand, the enhanced consistency branch improves prediction stability under in-domain perturbations and reduces representation drift. On the other hand, the prototype-guided counterfactual branch, together with prototype-calibrated decision, suppresses spurious-correlation reliance and improves the reliability of the final prediction. As a result, the method continues to deliver performance gains and improves diagnostic reliability even in a simpler label space.

### 5.3 Ablation experimental results

To evaluate the contributions of the major components of the proposed framework while preserving comparability with the original experimental protocol, the ablation study is still organized at the branch level. Specifically, the proposed framework continues to be decomposed into the consistency branch and the counterfactual branch. Here, the +DICR variant introduces the consistency regularization branch, the +EM variant introduces the enhanced counterfactual branch, and the full model combines both mechanisms with prototype-guided feature structuring and prototype-calibrated decision. Therefore, the branch-level ablation remains suitable for evaluating the contributions of the current design. The experimental results are reported in Table 6.

**TABLE 6:** Ablation study on two datasets (mean ± std). DICR denotes Domain-invariant Consistency Regularization, and EM denotes the counterfactual-related Enhancement Mechanism.

Dataset	Method	Acc	Precision	Recall	F1-score	AUC
Dataset1	Baseline	0.9412±0.0066	0.9329±0.0076	0.9219±0.0094	0.9259±0.0073	0.9927±0.0054
	+DICR	0.9637±0.0057	0.9489±0.0057	0.9465±0.0071	0.9525±0.0064	0.9951±0.0034
	+EM	0.9742±0.0052	0.9590±0.0044	0.9557±0.0056	0.9685±0.0047	0.9962±0.0028
	Ours	0.9823±0.0034	0.9676±0.0026	0.9740±0.0025	0.9805±0.0030	0.9982±0.0009
Dataset2	Baseline	0.9245±0.0082	0.9284±0.0068	0.9140±0.0090	0.9198±0.0077	0.9225±0.0055
	+DICR	0.9485±0.0050	0.9472±0.0043	0.9419±0.0060	0.9483±0.0049	0.9581±0.0035
	+EM	0.9593±0.0064	0.9614±0.0061	0.9604±0.0075	0.9593±0.0063	0.9854±0.0043
	Ours	0.9652±0.0033	0.9688±0.0026	0.9773±0.0025	0.9698±0.0030	0.9989±0.0009

The ablation results on both datasets indicate that the performance gains of the proposed framework come from clear module-level contributions rather than accidental effects from component stacking. On Dataset1, both + DICR and +EM improve all five metrics over the Baseline. Specifically, Acc/F1/AUC increase from 0.9412/0.9259/0.9927 in the Baseline to 0.9637/0.9525/0.9951 with +DICR and to 0.9742/0.9685/0.9962 with +EM, while the full model further reaches 0.9823/0.9805/0.9982. This demonstrates that the consistency regularization can effectively enhance model stability against window width/level fluctuations, noise, and mild imaging variations without changing the supervised task, resulting in a smoother decision boundary that is less affected by in-domain perturbations. The strong performance of +EM further indicates that the enhanced counterfactual branch can effectively suppress diagnostically irrelevant but highly correlated spurious cues. In the full framework, this branch further incorporates prototype-guided feature structuring and prototype-calibrated decision, which together guide the model to focus more on lesion evidence and yield sustained benefits in comprehensive metrics such as Acc, F1, and AUC. The full model achieves the best overall performance on Dataset1, highlighting the complementary relationship between stability constraints and counterfactual de-spuriousness: the former addresses the requirement that the same sample should be consistent across different views, while the latter addresses which evidence the model should rely on to make more reliable decisions.

In the binary classification setting of Dataset2, all three ablation variants outperform the Baseline, but their contribution patterns are slightly different. The + DICR variant improves Acc/F1/AUC from 0.9245/0.9198/0.9225 to 0.9485/0.9483/0.9581, indicating that consistency regularization alone already brings clear gains in stability and overall discriminability. The + EM variant further reaches 0.9593/0.9593/0.9854, suggesting that the enhanced counterfactual branch contributes more strongly to suppressing spurious correlations and improving threshold-independent discrimination in the binary setting. Considering the trends across both datasets, the full model achieves the highest Recall, F1, and AUC simultaneously, with Recall/F1/AUC reaching 0.9773/0.9698/0.9989 on Dataset2, indicating that the two proposed mechanisms not only improve average performance but also enhance positive-sample coverage and threshold-independent discriminative reliability, enabling more consistent diagnostic behavior under different data distributions and task difficulties.

### 5.4 t-SNE experimental results

**FIGURE 5:**
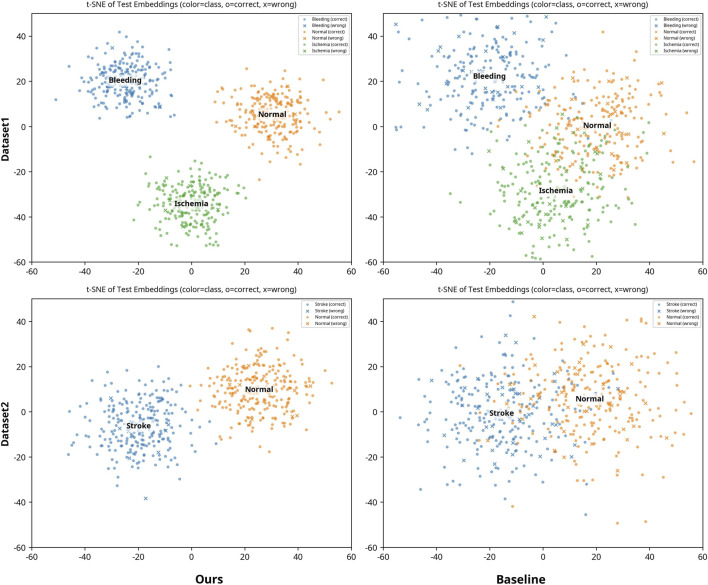
The method presented in this paper is compared with the baseline in the t-SNE visualization of feature embeddings on the test sets of two datasets (color represents the category, and dots/crosses represent correct/incorrect predictions, respectively).

To visually analyze the inter-class separability and intra-class compactness of the features learned by different methods, we perform a t-SNE visualization comparison between the proposed method and the Baseline on both datasets. Specifically, we extract the global feature vectors of test samples right before the classification head and project them into a two-dimensional space, so as to observe the clustering structure and overlap among different classes in the embedding space. This provides representation-level evidence beyond quantitative metrics. The visualization results are shown in Figure 5.

From the visualization results, we observe that the proposed method forms a more clearly separated and compact cluster structure on Dataset1 for the three-class task. The clustering boundaries among Bleeding, Ischemia, and Normal are more distinct, and the within-class distributions are more compact, with a smaller and more localized overlap region across classes. Moreover, the misclassified samples (marked by crosses) are more concentrated near the class transition regions rather than being broadly scattered, indicating that the learned representations are more discriminative and that the decision boundary is more stable. In addition, the feature refinement and confidence-aware aggregation mechanisms further promote intra-class compactness and inter-class separation, which is consistent with the clearer cluster structure observed in the t-SNE space. Similarly, in the binary setting of Dataset2, the proposed method exhibits a more pronounced separation trend between Stroke and Normal, with a narrower transition band and a clearer inter-class gap, suggesting a more consistent encoding of key stroke-related evidence and reduced confusion induced by imaging noise or in-domain perturbations. In contrast, the Baseline shows a more dispersed embedding distribution with a substantially larger central overlap region, and misclassified points are distributed more broadly within this mixed area, reflecting that its feature learning relies more on unstable cues and leads to a less well-defined class boundary.

### 5.5 Grad-cam experimental results

**FIGURE 6:**
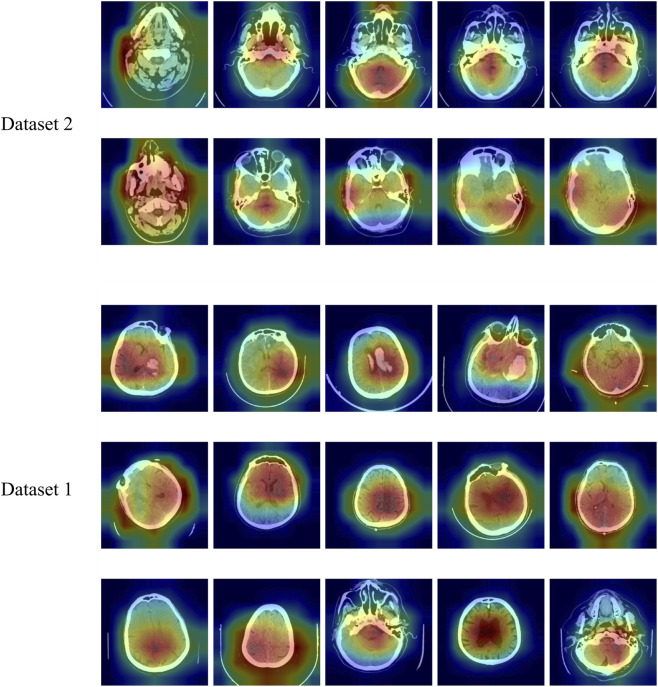
This figure shows an example of Grad-CAM visualization of the method presented in this paper on Dataset1 and Dataset2, with heatmaps overlaid on the original CT slices to characterize key areas of interest when making model decisions.

To further understand the basis of the model decisions from an interpretability perspective, we generate Grad-CAM heatmap visualizations for the proposed method on both datasets to examine which image regions the model attends to when making classification predictions. It should be noted that this section does not include comparisons with other methods; instead, we only present the visualization results of our approach on the two datasets to verify whether the attended regions exhibit consistent medical plausibility. In particular, for Dataset2, where pixel-level annotations are available, the Grad-CAM visualizations are qualitatively examined with reference to lesion locations to assess whether the highlighted regions remain clinically plausible. The visualization results are shown in Figure 6.

From the visualization results, it can be observed that the high-response regions of the model are predominantly confined to the intracranial area and frequently overlap lesion-containing regions or their surrounding tissues, rather than being widely distributed over the background outside the head region. At the same time, the attention maps are not always sharply localized, and some samples still exhibit relatively broad responses along the skull base, cortical boundary, or symmetric brain parenchyma, suggesting that the model also exploits a certain amount of contextual anatomical information. For Dataset1, the salient activations in most samples are distributed around abnormal-density regions, lesion-side hemispheres, or clinically suspicious intracranial structures, indicating that the learned representations exhibit a generally plausible but case-dependent degree of medical consistency and interpretability. This suggests that the model tends to rely on lesion-related cues for discrimination, although the exact focus may vary across bleeding, ischemia, and normal slices, especially when contrast is weak or lesion boundaries are unclear. For Dataset2, the heatmap responses are more often concentrated on the hemorrhage-containing side and adjacent peri-lesional tissues, and the highlighted regions are usually more compact and stable than those in Dataset1, which is consistent with the relatively simpler binary discrimination setting. Meanwhile, a subset of samples still shows diffuse or partially off-target activation, implying that complex anatomical background and imaging variations may introduce some interference; however, overall, these visualizations provide intuitive support for the plausibility that the model attention is largely aligned with lesion-relevant evidence while still retaining limited contextual dependence. This observation is consistent with the enhanced consistency branch and the prototype-guided counterfactual branch encouraging the model to preserve stable evidence focus and to rely more consistently on lesion-relevant cues rather than unstable artifact responses.

### 5.6 PR and ROC curves on two datasets

**FIGURE 7:**
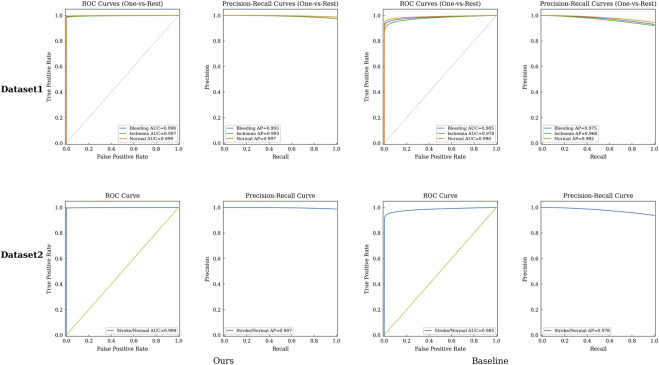
This figure shows the comparison of the ROC curves and PR curves of our proposed method and the baseline on two datasets.

To comprehensively evaluate the classifier’s discriminative ability from a threshold-independent perspective, this paper further plots the Precision-Recall (PR) curves and Receiver Operating Characteristic (ROC) curves for the baseline and the proposed method on two datasets. Compared to reporting only a single metric, the PR curve more sensitively reflects the precision-recall tradeoff under class imbalance conditions, while the ROC curve characterizes the overall discriminative properties under different discrimination thresholds. Based on the comparison of these two types of curves, the threshold-dependent discriminative behavior and precision-recall trade-off of the two methods can be more directly analyzed in both the multi-class and binary settings. The experimental results are shown in Figure 7.

From the curve shapes, it can be observed that the proposed method is overall closer to the ideal behavior on both datasets: the ROC curves lie closer to the upper-left corner and maintain a higher true positive rate in the low false positive rate region, while the PR curves preserve higher precision even at high-recall ranges, indicating more stable discriminative ability across different thresholds and a better precision–recall trade-off. For the one-vs-rest results on Dataset1, the ROC/PR curves of all classes show a stronger tendency to hug the upper envelope, with class-wise ROC AUCs reaching 0.998, 0.997, and 0.999 and PR APs reaching 0.993, 0.993, and 0.997 for Bleeding, Ischemia, and Normal, respectively. This suggests that the improvements are not driven by a single class but instead reflect more sufficient overall inter-class separation. For Dataset2, the proposed method exhibits an ROC AUC of 0.999 and a PR AP of 0.997, with both curves remaining very close to the ideal boundary, whereas the Baseline only reaches an ROC AUC of 0.985 and a PR AP of 0.978 and shows a more noticeable precision drop in the high-recall region. This is consistent with the objectives of enhancing prediction stability under in-domain perturbations via consistency constraints and suppressing spurious correlations via the counterfactual mechanism, thereby preserving a more reliable decision boundary even as the threshold varies.

### 5.7 Misclassification experiment results

**FIGURE 8:**
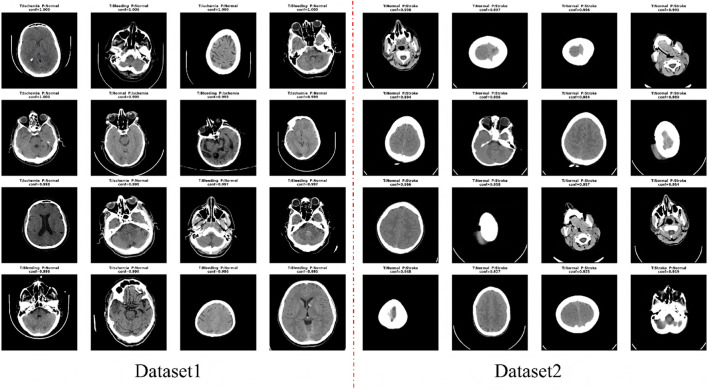
This figure shows the set of misclassified samples with the highest prediction confidence of our method on two datasets (each sample is labeled with the true class, predicted class, and corresponding confidence).

To further reveal potential failure modes in the actual inference process and aid subsequent improvements, this paper specifically analyzes the misclassification samples of our proposed method in the test set. It is important to emphasize that this section does not compare with other methods, but focuses solely on “high-confidence errors” where our model still misclassifies despite high prediction confidence, in order to more rigorously test its decision reliability. This analysis is particularly relevant to the proposed framework, because the prototype-calibrated decision mechanism is intended to suppress unreliable overconfident predictions rather than merely improve raw classification accuracy. Through visualization and attribution analysis of these samples, we can more intuitively pinpoint the potential factors leading to misclassification and provide a basis for data cleaning, label consistency checks, and model structure optimization. The experimental results are shown in Figure 8.

Analysis of the high-confidence misclassified samples shows that most errors occur in cases where the imaging evidence is intrinsically weak, locally ambiguous, or visually similar across categories. On Dataset1, most high-confidence errors correspond to Ischemia or Bleeding samples being misclassified as Normal, while only a few cases show other confusion patterns, such as mutual confusion between the two lesion classes. This pattern is likely related to low lesion contrast, limited lesion extent, blurred lesion boundaries, or lesion locations near artifact-prone regions such as the peripheral brain area and the skull base. Under such conditions, subtle abnormal density changes may appear visually similar to beam-hardening artifacts, noise patterns, or normal anatomical textures, causing the model to rely excessively on weak local responses and to produce biased predictions even with high confidence. On Dataset2, most high-confidence errors correspond to Normal samples being misclassified as Stroke, while only a small number show the reverse pattern. This suggests that the model remains sensitive to certain non-lesion structures with high-intensity responses, boundary-enhancement patterns, or strong contrast regions near the skull, and may incorrectly interpret these irrelevant cues as evidence of stroke. Overall, these high-confidence errors are unlikely to be purely random failures; rather, they more plausibly reflect systematic confusion caused by difficult case distributions, low signal-to-noise imaging conditions, variations in window width and window level, and local artifact interference. These findings further suggest several possible directions for improvement, including the introduction of finer lesion-region prior guidance, stronger hard-example reweighting or resampling strategies for ambiguous slices, and artifact-aware or edge-constrained augmentation methods to reduce the influence of non-lesion-dominant structures and improve robustness in clinically challenging cases.

### 5.8 Hyperparameter sensitivity experiment

We conduct all hyperparameter sensitivity studies on Dataset1 to isolate the effect of each design choice under a consistent data distribution and evaluation protocol. In the following, we vary one hyperparameter at a time while keeping all other settings fixed, and report the changes in Acc, Precision, Recall, and F1-Score. Although prototype-guided counterfactual refinement and confidence-aware calibration are incorporated into the current framework, the present sensitivity analysis focuses on the two most influential global control factors, namely, the intervention strength and the optimization step setting, so as to preserve comparability under a unified experimental protocol.

#### 5.8.1 Sensitivity to counterfactual intervention strength ε

**FIGURE 9:**
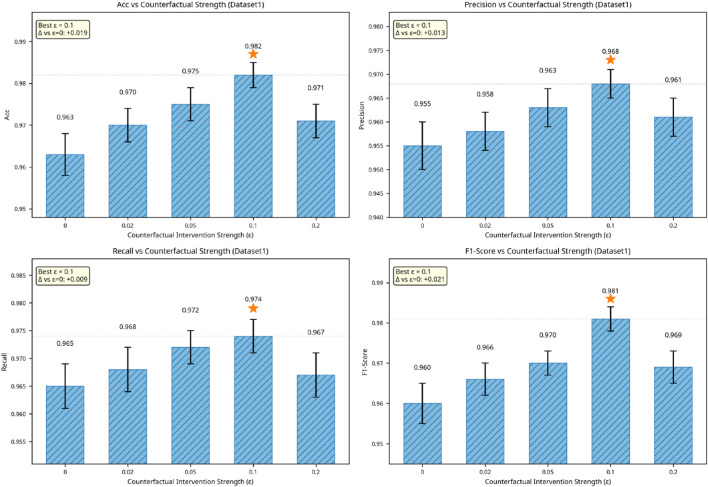
Hyperparameter sensitivity analysis of the counterfactual intervention intensity ε on Dataset1: Acc, Precision, Recall and F1-Score are statistically analyzed under different ε values, and a bar chart with error bars is provided to observe the trend of performance with intervention intensity and the optimal range.

This experiment investigates how the counterfactual intervention strength ε influences the stability of the learned decision boundary and the robustness of predictions on Dataset1. By sweeping ε across a set of values from weak to strong interventions, we examine whether moderate perturbations can better suppress spurious cues without damaging lesion-related evidence. The experimental results in Figure 9 show a clear non-monotonic trend, with overall performance improving steadily from ε = 0 to ε = 0.1 and then declining when ε is further increased to 0.2.

The results show that ε exhibits a clear “moderate-optimal” pattern: when ε increases from 0 to 0.02, 0.05, and 0.1, all four indicators improve overall and reach their best values at ε = 0.1, where Acc, Precision, Recall, and F1-Score rise to 0.982, 0.968, 0.974, and 0.981, respectively. Compared with the case of ε = 0, the gains at ε = 0.1 are approximately +0.019 in Acc, +0.013 in Precision, +0.009 in Recall, and +0.021 in F1-Score. This indicates that the counterfactual mechanism in this paper can effectively perturb spurious cues unrelated to lesions under moderate intervention, thereby improving the stability of the decision boundary and the reliability of generalization together with the consistency constraints. Moreover, the interval from ε = 0.05 to ε = 0.1 can be regarded as a relatively stable high-performance range, suggesting that the model remains robust under moderate intervention intensity. However, when the intervention is further increased to ε = 0.2, the indicators decline, indicating that excessive intervention will introduce damage to effective discriminative information related to lesions or produce excessive distribution shifts, making it difficult for the model to maintain the causal/invariant features learned from the structural constraints, thus weakening the design goal of “suppressing spurious correlations and preserving key evidence” of the proposed method.

#### 5.8.2 Sensitivity to learning rate lr

**FIGURE 10:**
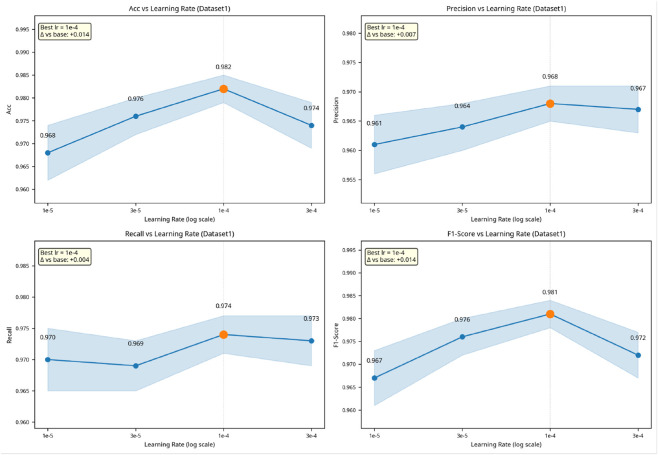
Hyperparameter sensitivity analysis of learning rate lr on Dataset1: Curves of Acc, Precision, Recall and F1-Score are plotted under multiple lr settings on a logarithmic scale, and the fluctuation range of multiple runs is characterized by error bands to evaluate optimization stability and appropriate training step size range.

This experiment analyzes the impact of the learning rate lr on optimization dynamics and final performance on Dataset1. We evaluate a log-spaced range of lr values to characterize under-training at overly small steps and potential instability at overly large steps, thereby identifying a stable operating region for the proposed training objective. The experimental results are shown in Figure 10.

The learning rate sensitivity results show that performance first improves as lr increases from 1e-5 to 1e-4 and then slightly declines at 3e-4, indicating the existence of a relatively favorable intermediate range. lr mainly affects the optimization dynamics and convergence quality of the joint objective through the coordination between classification supervision, consistency regularization, and counterfactual refinement: too small lr often leads to insufficient collaborative updates between the constraint terms and the main classification objective, resulting in lower overall performance or slow improvement. As lr increases to a moderate level, the performance indicators reach better levels and become more stable, with the best overall results achieved at lr = 1e-4. Specifically, Acc, Precision, Recall, and F1-Score reach 0.982, 0.968, 0.974, and 0.981, respectively, at lr = 1e-4, corresponding to gains of about +0.014, +0.007, +0.004, and +0.014 over the 1e-5 setting. This indicates that a moderate step size can more effectively coordinate the updates of different objective terms and help the model reach a better balance between discriminability and stability. The range from 3e-5 to 1e-4 can therefore be regarded as a relatively suitable operating interval. However, when lr increases further to 3e-4, Acc and F1-Score decrease noticeably, while Precision and Recall remain comparatively high, suggesting that an excessively large update amplitude may weaken the stabilizing effect of structural constraints on the representation space and make training more susceptible to noisy gradients or hard example perturbations. This verifies the requirement of the proposed method for a properly controlled learning rate to balance discriminability and constraint stability.

### 5.9 Visualization of lesion and artifact feature separation

**FIGURE 11:**
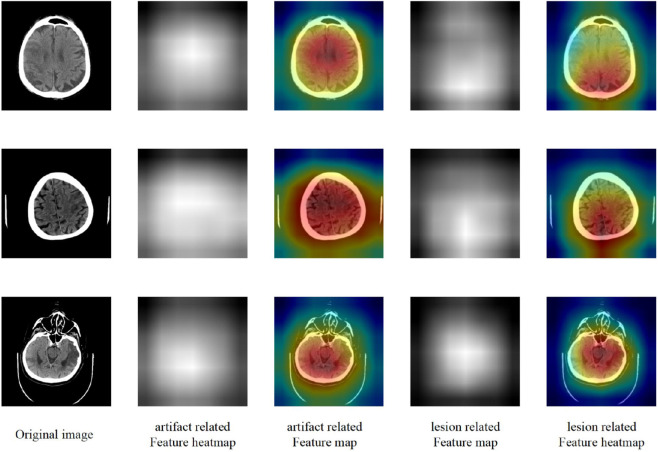
Visualization of feature decomposition by the proposed counterfactual mechanism, where the separated artifact-related and lesion-related responses are shown for representative stroke CT slices to illustrate the spatial distinction between interference cues and clinically meaningful lesion evidence.

To provide a more intuitive understanding of lesion–artifact disentanglement in the proposed framework, this study further presents a visual analysis of lesion-related and artifact-related feature responses in stroke CT slices. By separately examining the spatial activation patterns of the two feature components, the analysis helps reveal whether the model can distinguish clinically meaningful lesion evidence from interference cues introduced by bony structures and imaging artifacts. The experimental results are shown in Figure 11.

From the visualization results, the feature decomposition demonstrates a visually meaningful disentanglement tendency and a relatively clear functional separation pattern. The artifact-related branch exhibits broader and more peripheral responses, mainly concentrating on the high-intensity skull boundaries, the scanning field margins, and some regions reflecting global intensity variation, which indicates that this branch is more inclined to capture non-lesion cues associated with imaging conditions, bony structures, or artifact interference. In contrast, the lesion-related branch produces stronger responses within the intracranial parenchymal region and, in several cases, shows more localized emphasis on asymmetric or suspicious intracranial areas, while the activations in the background and peripheral irrelevant areas are relatively suppressed. Across the three representative samples, the two types of heatmaps show a consistent but not completely isolated spatial division pattern, suggesting that the observed lesion-artifact separation is jointly supported by prototype-enhanced representation learning and the subsequent counterfactual intervention, rather than by counterfactual decomposition alone. This indicates that the proposed framework does not simply redistribute the original representation, but organizes feature responses in a more structured manner, thereby providing a clearer and more stable basis for subsequent classification.

## 6 Conclusion

### 6.1 Main findings

This study addresses the task of automatic stroke recognition from single CT slices and proposes a unified slice-level classification framework that integrates consistency regularization, counterfactual suppression, and multi-branch feature refinement. The method is developed to mitigate diagnostic errors caused by lesion heterogeneity, large contrast variation, and interference from bony structures and imaging artifacts in clinical CT images. Experimental results on two datasets demonstrate that the proposed framework achieves stable and consistent improvements across the major evaluation metrics, confirming its effectiveness for stroke CT slice recognition.

### 6.2 Method effectiveness and interpretation

From a methodological perspective, the consistency regularization module encourages the model to maintain stable predictive behavior under perturbations and structure-preserving transformations, thereby improving its ability to focus on lesion-relevant evidence. At the same time, the counterfactual branch suppresses potentially misleading cues while refining branch-wise evidence through confidence-aware aggregation, which reduces incidental reliance on non-lesion regions and helps alleviate unreliable overconfident predictions. Furthermore, the visualization results show that the proposed framework yields attention responses that are more concentrated in clinically meaningful regions, while the lesion-related and artifact-related feature responses exhibit a clearer functional separation pattern, providing additional support for its effectiveness in suppressing spurious correlations while preserving useful discriminative information.

## Data Availability

The original contributions presented in the study are included in the article/supplementary material, further inquiries can be directed to the corresponding author.
